# Supplementary material for: A portable, ultra-low cost, open-source, pedal-controlled microinjector for laboratory use
Source: PLoS One. 2026 May 27;21(5):e0347487. doi: 10.1371/journal.pone.0347487 (PMC13215527; doi:10.1371/journal.pone.0347487)
Supplement: S1 File — (ZIP) [file pone.0347487.s004.zip › S4 Readme.pdf]

S4 File: Source Data and Code

This compressed archive (.zip) contains the raw datasets and Python scripts used to generate the quantitative analyses and plots presented in the manuscript. Included source data files contain measurements of microinjector performance, including droplet volumes from pulse-controlled injections, static pressure measurements, needle diameter measurements, and viscosity measurements of injection solutions. Data files are provided in comma-separated values (.csv) format. The archive also includes Python scripts used to process the datasets and generate the corresponding plots used in the manuscript. These scripts produce categorical and continuous analyses of injection performance, including relationships between pulse duration, pressure settings, and injection variability (Figs. 5-6). All scripts are written for Python 3 and rely on standard scientific Python libraries (e.g., NumPy, pandas, and Matplotlib). Usage:

```
python3 generate_continuous_plots_pressure.py "Static Pressure Measurements.csv"
python3 generate_categorical_plots_all_data.py "Microinjector Measured Injections.csv"
python3 generate_continuous_plots_errors.py "Microinjector Measured Injections.csv"
```
