# Supplementary material for: A portable, ultra-low cost, open-source, pedal-controlled microinjector for laboratory use
Source: PLoS One. 2026 May 27;21(5):e0347487. doi: 10.1371/journal.pone.0347487 (PMC13215527; doi:10.1371/journal.pone.0347487)
Supplement: Attachment — Submitted filename: 2026-03 Reviewer Response rev3.docx [file pone.0347487.s005.docx]

**We thank the Academic Editor and the reviewers for their thoughtful and constructive feedback. The comments have significantly helped us improve the clarity and usefulness of the manuscript. Below we respond to each point in detail and describe the revisions made.**

1.Please ensure that your manuscript meets PLOS ONE's style requirements, including those for file naming. The PLOS ONE style templates can be found at

https://journals.plos.org/plosone/s/file?id=wjVg/PLOSOne_formatting_sample_main_body.pdf and

<https://journals.plos.org/plosone/s/file?id=ba62/PLOSOne_formatting_sample_title_authors_affiliations.pdf>

2. Please note that funding information should not appear in any section or other areas of your manuscript. We will only publish funding information present in the Funding Statement section of the online submission form. Please remove any funding-related text from the manuscript.

3. We note that the grant information you provided in the ‘Funding Information’ and ‘Financial Disclosure’ sections do not match.

When you resubmit, please ensure that you provide the correct grant numbers for the awards you received for your study in the ‘Funding Information’ section.

4. Please include captions for your Supporting Information files at the end of your manuscript, and update any in-text citations to match accordingly. Please see our Supporting Information guidelines for more information: <http://journals.plos.org/plosone/s/supporting-information>.

5. We are unable to open your Supporting Information file generate_categorical_plots_all_data.py and generate_continuous_plots_errors.py. Please kindly revise as necessary and re-upload.

6. If the reviewer comments include a recommendation to cite specific previously published works, please review and evaluate these publications to determine whether they are relevant and should be cited. There is no requirement to cite these works unless the editor has indicated otherwise.

**1. The manuscript has been reformatted for compliance with PLOS ONE style. Figure legends are now placed in the manuscript immediately after their first citation, Supporting Information captions are included at the end of the manuscript, and figures are uploaded as separate files in the submission system.**

**2. All funding-related language has been removed from the manuscript text. Funding information is now provided exclusively through the Funding Statement in the submission system, as required by PLOS ONE guidelines.**

**3. The Funding Information has been reviewed and corrected within the Editorial Manager submission system to ensure that all grant numbers and funding sources are consistent. That said, we were not able to update the existing Financial Disclosure statement within Editorial Manager, so we have provided this as an add-on “Other” file on the attachment page in the submission system.**

**4. Supporting Information captions have now been added to the end of the manuscript, and the corresponding in-text citations have been updated accordingly.**

**5. We apologize for the oversight, as the original python scripts were designed to be used without melted data tables, but the melted tables were the ones provided to accompany them. The Python scripts used to generate the analysis plots have been re-uploaded and paired with the correct source data files, and instructions for their use are now in the Supporting Information Legend for S6 File corresponding to these.**

**6. We have reviewed the suggested references and incorporated relevant citations discussing recent advances in small-volume and microinjection-based drug delivery technologies. These citations have been added to the Introduction to provide additional context for readers interested in related injection technologies.**

Reviewers' comments:

Reviewer's Responses to Questions

**Comments to the Author**

1. Is the manuscript technically sound, and do the data support the conclusions?

The manuscript must describe a technically sound piece of scientific research with data that supports the conclusions. Experiments must have been conducted rigorously, with appropriate controls, replication, and sample sizes. The conclusions must be drawn appropriately based on the data presented.

Reviewer #1: Yes

Reviewer #2: Yes

2. Has the statistical analysis been performed appropriately and rigorously?

Reviewer #1: Yes

Reviewer #2: Yes

3. Have the authors made all data underlying the findings in their manuscript fully available?

The [PLOS Data policy](http://www.plosone.org/static/policies.action#sharing) requires authors to make all data underlying the findings described in their manuscript fully available without restriction, with rare exception (please refer to the Data Availability Statement in the manuscript PDF file). The data should be provided as part of the manuscript or its supporting information, or deposited to a public repository. For example, in addition to summary statistics, the data points behind means, medians and variance measures should be available. If there are restrictions on publicly sharing data—e.g. participant privacy or use of data from a third party—those must be specified.

Reviewer #1: Yes

Reviewer #2: Yes

4. Is the manuscript presented in an intelligible fashion and written in standard English?

PLOS ONE does not copyedit accepted manuscripts, so the language in submitted articles must be clear, correct, and unambiguous. Any typographical or grammatical errors should be corrected at revision, so please note any specific errors here.

Reviewer #1: Yes

Reviewer #2: Yes

5. Review Comments to the Author

Please use the space provided to explain your answers to the questions above. You may also include additional comments for the author, including concerns about dual publication, research ethics, or publication ethics. (Please upload your review as an attachment if it exceeds 20,000 characters)

Reviewer #1: This report concerns the manuscript entitled “A portable, ultra-low cost, open-source, pedal-controlled microinjector for laboratory use”.

After reading the manuscript, I believe that this work has very useful information and it should be published after some considerations that I hope will enhance the impact and relevance. The authors provide a very well written manuscript and clear instructions on how to build a cost-effective device.

In the Introduction part, can some details be given about the delivery timescale, and penetration depth of the envisaged injections? Assuming that the system is not agnostic to the fluid type, can a list of fluids with physicochemical properties range be given to educate the future users? E.g., around line 135, Typical injection/aspiration times, at least for water? Depending on materials/fluid - would it work with non-aqueous liquids?

**We thank Reviewer #1 for their positive evaluation and helpful suggestions to improve the clarity and usability of the manuscript.**

**We have added a brief description of typical injection timescales to the Methods section describing device operation. For low-viscosity aqueous solutions, aspiration events typically occur over ~1–3 s, while pulse-controlled injection events typically occur over ~5–100 ms depending on the selected pulse duration and pressure setting. Because penetration depth depends primarily on the needle geometry and biological preparation rather than the injector hardware itself, this parameter is not fixed by the device and was therefore clarified in the revised text.**

**A paragraph describing supported fluid types has been added to the Methods section. The injector is compatible with a range of injectates including aqueous buffers, plasmid solutions, viral suspensions, and low-viscosity oils. The revised manuscript also clarifies that highly viscous fluids can be dispensed but may require higher pressure settings and longer pulse durations.**

When stating that inspection/checking for leaks, is it meant by optical (eye)inspection for droplets? Or ‘gas’ leaks … how?

**The manuscript has been revised to clarify that leak testing refers to visual inspection of tubing junctions under pressure. Additional details are provided in a Troubleshooting section that has been moved to Materials and Methods.**

Is it possible to say something about the use biodegradable materials? What happens after usage of components, such as the needle that has been in contact with different substrates?
Could the authors discuss some considerations about recycling/sustainability?

**Thank you for this important consideration, especially when parts costs are quite low compared with the commercial devices our microinjector is intended to replace. We have added a section “Materials, disposal, and sustainability considerations” in the Discussion section, to address reader concerns about one-time usage of this device, and how it may fare comparably or better to those commercial systems.**

Near line 160, Are there references about red cabbage as biological surrogate?

**Thank you for this point of clarification. Our description of red cabbage extract as a biological surrogate was derived purely from our experiential handling and injections with plasmid or CRISPR solutions, and red cabbage extract solutions. Notably, red cabbage extract in water does retain some tiny particulates that have gross performance characteristics during microinjection that reminded us of handling actual DNA or CRISPR solutions. However, we have not characterized these particulates in any scientifically rigorous manner, so we have tempered the language in the Materials and Methods section to what is factually known without speculation.**

Near line 165, can the authors be more specific about the lamp oil? E.g., manufacturer or cP or other ‘units’?

**Yes, the paraffin lamp oil used now has a manufacturer (Ner Candles, Miami FL) listed in the Materials and Methods section. Viscosity data for this brand is not explicitly listed, though paraffin lamp oils of this type generally range between 1 and 5 cP.**

Line 170, are the large bubbles observed due to air entrainment during droplet impact on the lamp oil? Some videos or images would be very helpful, e.g., around line 185, Visualizations of the injection into the lamp oil?

**Thank you for mentioning this. This was quite an interesting observation during experiments prior to our initial submission. Such injections were performed into center well dishes for maximal ease and accessibility for the operator. However, we could not replicate the fragmentation when larger vessels (i.e. 10 cm Petri Dish) were used. Instead high pressures caused droplets to eject quickly and travel away from the needle tip (see final injections in new S5 Video). Therefore, we concluded that the droplet fragmentation was likely a result of fast-moving, large droplets impacting the side walls of the vessel, not a pure fluidics phenomenon. We have altered the language in the manuscript around this to better reflect our continued experience with quantification of microinjection.**

Line 190, Is it possible to show pictures of how the device is ‘held’ by somebody’s hands.
Ergonomic aspects not needed at this stage, but could be useful to say something about it.

**Thank you for raising this issue. The operation of the microinjector, both in terms of its user interface, pressure control, and foot actuation, are now shown in a new S4 Video. Reproducing droplets with the pulse controller is shown in a new S5 Video. We feel these additions strengthen the manuscript significantly by not only placing visuals for reader context, but also by showing how experimental observations were obtained.**

Line 245, Any risks of cross-contamination in between changing needle, or vapor residues in the tubes ?

**Yes, and this is actually a major concern with virtually all commercial microinjectors as well. In all setups, cross contamination is typically managed by frequent glass needle changeovers, though plastic/metal needle holder components and tubing are rarely replaced due to costs and limitations in acquiring proprietary components that make up those apparatuses. We have added a paragraph in the Discussion to highlight this issue, and to describe cross-contamination as easier to mitigate with our custom home-built needle holder, which is comprised of cheap commodity parts that are either autoclavable or facilely replaceable.**

Line 270, Can some explanation be given, and perhaps backed up by literature about fluid dynamics of such process of droplet/jet ejection instabilities?A short qualitative discussion of droplet formation and instability regimes has been added to the Discussion section. Because the primary goal of the manuscript is to describe the device hardware rather than perform a detailed fluid dynamics study, this discussion remains qualitative.

Line 290, Is it possible to provide some fluid dynamics considerations/equations that relate the fluidic properties with the pressure differences and observables: perhaps simple Bernoulli does a good job.

**A discussion of droplet formation and stability has been added to the Discussion section, and we have added a new section in Discussion on fluid dynamics theory. We have reviewed and cited studies from microfluidics and physics literature that relate needle diameter, pressure, and jet ejection stability. Because droplet formation in our microinjector system depends strongly on a wide variety of intrinsic and extrinsic factors including needle geometry and experimental setup, we opted to present empirical measurements rather than theoretical predictions. Nonetheless, fluid dynamics theory and flow principles of droplets vs. jet stream provide outstanding context and concrete explanations for what we have observed and presented in this manuscript, and we are excited to add this to the Discussion.**

Line 325, Is it possible to provide a video showing apsire/inject operation?
How noisy it is to operate?

**We thank the reviewer for this excellent suggestion, and have added S4 Video to demonstrate operation of the microinjector, including aspirate and inject cycles. Moreover, we have added an additional Figure 5 to demonstrate reproducibility of droplet formation using the pulse controller. We also directly measure pressure in the tubing to best understand how pulse width modulation (PWM) of the motor controller directly relates to and controls injection or aspiration pressure.**

Line 330, what are the estimated shear forces that the injectate will experience in the inject/aspire process?
Some injectable fluids may be sensitive to shear. How robust are plasmid and other molecules envisaged by the authors?

**We thank the reviewer for pointing this out. We have added a new Discussion section to describe in qualitative terms the advantages of a diaphragm pump driven injection system for minimizing shearing forces, particularly when compared with systems utilizing rigid components brought into moving contact with the injectate (i.e. Hamilton syringe). We hope this will address the reviewer’s astute concerns on this important topic.**

Line 370, Can a citation be given about agonist/antagonist effects?

**Thank you for pointing out this oversight in our descriptions of potential applications. The agonist/antagonist idea was based on work from the 2000s, using mostly injection with standard needles or even systemically administered agents. We have adjusted the sentence accordingly to not inappropriately narrow scope to agonist-antagonist interactions but to highlight the power of localized delivery of drugs or genetic manipulations. References are now included.**

Suggestions:
Recent work on small volume (Drug delivery) injection methods has been covered in recent reviews:
1. Schoppink, Jelle, and David Fernandez Rivas. "Jet injectors: Perspectives for small volume delivery with lasers." Advanced drug delivery reviews 182 (2022): 114109.
2. Phatale, Vivek, et al. "Overcoming skin barriers through advanced transdermal drug delivery approaches." Journal of controlled release 351 (2022): 361-380.

**In the Introduction section, we have broadened the discussion to make the case for microinjection as a subset solution within a larger domain of small precision delivery systems. Thank you for these suggested readings, that we have assimilated.**

Reviewer #2: This work titled "A portable, ultra-low cost, open-source, pedal-controlled microinjector for laboratory use" reports a low-cost, open-source microinjector that pairs a compact, PWM-driven diaphragm pump with bidirectional valve control and footswitch actuation to enable rapid, controllable microinjection. Overall, the work is practical and interesting, demonstrating considerable technical skill from the researchers. However, several issues should be addressed before the manuscript can be considered for publication.

**We thank Reviewer #2 for their constructive suggestions to improve the clarity of the device description.**

Comment 1: The air pump and solenoid valve are the core components of this injector. It is recommended to provide a more detailed introduction to their working principle and the differences in switching between suction/injection modes.

**We thank the reviewer for this feedback. Indeed, when we invited others to test-build our microinjector we encountered a common problem that the solenoid valves connections were not always properly orientated, which is essential given the mechanism of the selectable bi-directional flow. We have expanded the Methods section to include a clearer explanation of the diaphragm pump and bidirectional valve system. The revised text explains how airflow is redirected to achieve aspiration and injection modes and how the footswitch activates the pump during operation.**

Comment 2: In lines 34 and 77, the authors mention that the system is capable of "enabling multiplexed injections or rapid switching between reagents within a single experiment." However, the Materials and Methods section and Results section do not describe how this functionality is implemented or demonstrate its use. The author needs to clarify or experimentally verify this feature.

**We have revised the manuscript throughout to clarify that multiplexed injections refer to sequential loading and dispensing of multiple reagents through the same needle during a single experiment. The Methods section now explicitly describes this workflow.**

Comment 3: In line 359-364, the authors state that the injector was applied to mouse embryo injections and cite references [7, 21–24]. Yet, none of these references appear to have used the injector described in this work. Suggest the author to supplement relevant supporting materials.

**Thank you for raising this important issue; after all, the injector is a tool to perform work, and is only as good as the work it does. To clarify, we have not *published* any papers using this injector, but have cited several instances both in our hands-on experience and in literature review where such an injector could be used. Still, our injector has been used in our laboratory for *in vivo* delivery experiments such as intra-oviductal CRISPR reagent delivery using iGONAD-style approaches – these are presently unpublished (see Fig. R1 for demonstration). As this manuscript is centered on the injector hardware itself and its synthetic performance, biological outcomes were not presented as we felt they would detract from that focus. If the reviewers feel it would substantially strengthen this manuscript, we could include some of this basic iGONAD verification data and/or images of injected oviducts – but these are neither novel (there are copious published examples of iGONAD demonstrations) nor directly within the current scope of this work at present time.**

Figure R1: Our laboratory has adopted intra-oviductal CRISPR injection and electroporation (iGONAD), which permits the rapid creation of genetically modified mice (or rats) in a single step. We employed iGONAD to correct the mutant *Tyr* gene in albino mice, which restores pigmentation (A). Next, we generated GFP-Klf2 fluorescent reporter mice (B).

Comment 4: For a study focused on the microinjection device, a key aspect is demonstrating its effectiveness. The authors are encouraged to supplement a video demonstration of the system in operation.

**We appreciate and agree wholeheartedly with the reviewer’s thoughts and ideas here. We have included S4 and S5 Videos – S4 Video in particular – to demonstrate the typical use (and ease of use) and operation of this microinjector. We hope this will inspire readers and encourage adoption of this open hardware.**

Comment 5: Suggest adding demonstration experiments for practical application scenarios, such as microinjection of certain zebrafish or mouse embryos.

**We thank the reviewer for raising this possibility and suggestion. We indeed appreciate that this injector is intended to perform laboratory tasks and not be an end in itself. However, as we described above, we wanted to center this study on the injector hardware and its synthetic performance characteristics, which we have further enhanced and strengthened following reviewer feedback. We are happy to consider addition of mouse iGONAD verification as described above, and/or depict some images of injections if the reviewer feels unconfident in real biological applicability given our synthetic data. Nonetheless, we did not include such data at this stage given our comfort with and desire to maintain the manuscript’s present scope and breadth.**

Comment 6: A critical technical concern is how consistent output volume is maintained when needle diameter varies, which can occur when using a pipette puller to prepare glass needles.

**We thank the reviewer for this critical feedback. The manuscript now explicitly states that injection volume depends on needle tip geometry. Furthermore, we have made direct pressure measurements and examined various needle properties under our experimental conditions, which is now shown in new Figure 5. Following this valuable data, we better understand the relationship between motor control duty cycle and delivered injection pressure. The Results and Discussion sections also emphasize that users should calibrate pulse duration versus delivered volume for each needle type.**

6. PLOS authors have the option to publish the peer review history of their article ([what does this mean?](https://journals.plos.org/plosone/s/editorial-and-peer-review-process#loc-peer-review-history)). If published, this will include your full peer review and any attached files.


If you choose “no”, your identity will remain anonymous but your review may still be made public.


**Do you want your identity to be public for this peer review?** For information about this choice, including consent withdrawal, please see our [Privacy Policy](https://www.plos.org/privacy-policy).

Reviewer #1: No

Reviewer #2: No
